# Sodium Thiosulfate Prevents Chondrocyte Mineralization and Reduces the Severity of Murine Osteoarthritis

**DOI:** 10.1371/journal.pone.0158196

**Published:** 2016-07-08

**Authors:** Sonia Nasi, Hang-Korng Ea, Frédéric Lioté, Alexander So, Nathalie Busso

**Affiliations:** 1 Service of Rheumatology, Department of Musculoskeletal Medicine, Centre Hospitalier Universitaire Vaudois and University of Lausanne, Lausanne, Switzerland; 2 Hospital Lariboisière, Service of Rheumatology, University School of Medicine, Paris VII, Paris, France; Faculté de médecine de Nantes, FRANCE

## Abstract

**Objectives:**

Calcium-containing crystals participate in the pathogenesis of OA. Sodium thiosulfate (STS) has been shown to be an effective treatment in calcification disorders such as calciphylaxis and vascular calcification. This study investigated the effects and mechanisms of action of STS in a murine model of OA and in chondrocyte calcification.

**Methods:**

Hydroxyapatite (HA) crystals-stimulated murine chondrocytes and macrophages were treated with STS. Mineralization and cellular production of IL-6, MCP-1 and reactive oxygen species (ROS) were assayed. STS's effects on genes involved in calcification, inflammation and cartilage matrix degradation were studied by RT-PCR. STS was administered in the menisectomy model of murine OA, and the effect on periarticular calcific deposits and cartilage degeneration was investigated by micro-CT-scan and histology.

**Results:**

*In vitro*, STS prevented in a dose-dependent manner calcium crystal deposition in chondrocytes and inhibited *Annexin V* gene expression. In addition, there was a reduction in crystal-induced IL-6 and MCP-1 production. STS also had an antioxidant effect, diminished HA-induced ROS generation and abrogated HA-induced catabolic responses in chondrocytes. *In vivo*, administration of STS reduced the histological severity of OA, by limiting the size of new periarticular calcific deposits and reducing the severity of cartilage damage.

**Conclusions:**

STS reduces the severity of periarticular calcification and cartilage damage in an animal model of OA via its effects on chondrocyte mineralization and its attenuation of crystal-induced inflammation as well as catabolic enzymes and ROS generation. Our study suggests that STS may be a disease-modifying drug in crystal-associated OA.

## Introduction

Osteoarthritis (OA) is a joint disease, characterized by degeneration of articular cartilage, osteophytes formation and synovial lining hyperplasia [1, 2]. OA affects millions of people worldwide and is the most prevalent cause of disability in the elderly. As such, it represents a major medical, economic and social challenge for aging societies. Current OA treatment strategies aim at addressing the symptomatic consequences of the disease rather than its underlying causes and surgical joint replacement still constitutes the final therapeutic option in the management of OA. Therapeutic alternatives that could affect the progression of the underlying disease and stop or reverse OA progression would represent a major breakthrough.

A potentially important but still poorly studied feature of OA is its intra-articular calcium-containing crystals deposition. Crystal deposits were identified in half of OA synovial fluids [3] and in all OA cartilage samples from knee or hip joint replacement surgery [4]. *In vitro*, calcium-containing crystals can induce mitogenesis as well as catabolic and inflammatory responses [5–12]. Basic calcium phosphate (BCP) crystals, —which encompass, hydroxyapatite (HA), carbonated apatite (CA) and octacalcium phosphate (OCP) crystals—and calcium pyrophosphate dihydrate (CPPD) are the two most common forms of crystals found in OA joint structures. The importance of BCP crystals in OA pathogenesis was recently demonstrated through intra-articular injection of BCP crystals which led to a chronic arthropathy in mice characterized by low grade inflammation and cartilage degradation [13]. More recently, in menisectomized mice, a validated model of OA, we observed an accumulation of joint calcific deposits in the form of BCP crystals. In this model, crystal deposition correlated with cartilage degradation and IL-6 expression [14]. Thus, compounds that interfere with crystal deposition ought to bring therapeutic benefit in the context of OA.

Different agents have been used to prevent calcification or its consequences in OA: phosphocitrate was shown to block the deleterious cellular responses stimulated by calcium-containing crystals and to prevent hydroxyapatite crystal formation with promising disease-modifying effects in both crystal-associated [15] and noncrystal-associated [16] OA models. In contrast, bisphosphonates, which can induce complete dissolution of arterial calcification secondary to the mutation in the ectonucleotide pyrophosphatase/phosphodiesterase (PC-1) gene [17], failed to inhibit the development of experimental OA [18]. In here we assess the therapeutic effect of sodium thiosulfate (STS, Na_2_S_2_O_3_) in the context of OA. STS was initially used to prevent or treat various conditions, such as cyanide poisoning, carboplatin, cisplatin-induced nephrotoxicity and renal lithiasis. Later it prove effective in calcium-associated conditions such as calciphylaxis (calcific uremic arteriolopathy), a rare but an important cause of morbidity and mortality in patients with chronic and end-stage kidney disease [19] and vascular calcification in uremic rats [20]. In the latter study, the prevention of calcification by STS can be explained by the combined calcium-chelating and acidosis-inducing properties, which then cause hypercalciuria and a negative net calcium balance. Interestingly, STS can increase the availability of locally acting calcification inhibitors such as matrix Gla protein [20]. In addition, STS has been reported to have antioxidant properties by acting as a free radical scavenger [21, 22]. Based on these evidences, we hypothesized that STS could decrease intra-articular calcium-containing crystals deposition and OA severity. In this work, we provide new insights in the underlying effect of STS in chondrocytes. In addition, we provide evidence that STS could be a possible disease-modifying drug for crystal-associated OA therapy.

## Materials and Methods

### Mice and induction of experimental osteoarthritis

Female C57BL/6 mice (8–10 weeks old) were purchased from Charles River. All animals were specific-pathogen-free and kept in a temperature-controlled environment in a ventilated rack with a 12:12-h light:dark cycle. Food and water were given ad libitum. Mice were anesthetized via continuous isoflurane inhalation by using an automatic delivery system that provides a mixture of isoflurane and oxygen continuously. Surgical tolerance was defined as absent pedal withdrawal reflex response. Knee joint instability was induced surgically by partial medial menisectomy (MNX) of the right knee, whereas the contralateral knee was sham-operated as control [23]. After surgery, all animals were allowed to spontaneously breathe room air and were placed under a warming light. As approved by the institutional animal care and use committee, 1.3mg/ml Paracetamol (Dafalgan, Bristol-Myers Squibb SA) was administered in drinking water the first two days after surgery. Animals’ behavious was monitored every day following menisectomy. Mice were injected i.p with 0.5 ml of STS (0.4g/Kg of body weight) or of vehicle (PBS), three times per week and for all the duration of the experiment. 2 months after MNX, mice were sacrificed via CO_2_ inhalation, knee dissected and fixed in 10% formalin.

### MicroCT- scan

MicroCT-scan was performed as described before [14]. New formed calcific deposits, at the place of the removed medial meniscus, were considered as Volumes Of Interest (VOI) for the quantitative analysis of new formation volume (mm^3^) and new formation crystal content (μg).

### Mouse knee histology and immunohistochemistry

After microCT-scan images acquisition, knees were decalcified in 5% formic acid, dehydrated, and embedded in paraffin. Sagittal sections (6 μm) of the knee medial compartment (3 sections/mouse spaced 70 μM apart) were stained with Safranin-O and counterstained with fast green/iron hematoxylin. Histological scorings (cartilage damage and Safranin-O loss) were assessed using the OARSI score [24], by two observers blinded with regard to the mice groups.

### Calcium phosphate crystals

Hydroxyapatite (HA) crystals were synthesized and characterized as previously described [25]. HA crystals were sterilized by gamma-radiation and pyrogen-free (≤ 0.01 EU/10 mg- by Limulus amebocyte cell lysate assay). Prior to experimentation, crystals were resuspended in sterile PBS and sonicated for 5 min.

### Bone marrow derived macrophage (BMDM) preparation

Bone marrow cells were isolated from the tibia and femur of C57BL/6 mice. For their differentiation into BMDM, the extracted cells were incubated for 7 days in Petri dishes with 30% L929 conditioned media (source of M-CSF) and 20% FBS in Dulbecco’s Modified Eagle Media (DMEM). The resulting BMDM were detached using cold PBS, and plated for stimulation experiments in either complete DMEM medium (Gibco), (10% FBS and 1% Penicillin Streptomycin (Sigma)), or incomplete DMEM (1% Penicillin Streptomycin only). For BMDM crystal formation analysis, cells were cultivated up to 7 days in complete BJGb medium (Gibco) (10% FBS, 50 μg/ml L-ascorbic acid 2-phosphate, 20 mM β-glycerol phosphate and 1% Penicillin Streptomycin). Medium was changed at day 3 of culture.

### Joint chondrocyte (CHs) preparation

Chondrocytes were isolated from C57BL/6 mice as described previously, with slight modifications [26]. Briefly, the joint cartilage (articular and epiphyseal) was harvested from the knee and hip joints of mice aged between 4–6 days. The cartilage was degraded by a three step digestion process by using decreasing concentrations of Liberase (Roche). The day after, the digested tissue was passed through a 70μm filter (BD biosciences) to obtain immature chondrocytes. The cells were plated into a culture plate at high density (3.5x10^4^ cells/cm^2^) and amplified for 7 days in complete DMEM (10% FBS, 1% Penicillin Streptomycin). Prior to crystal stimulation experiments, cells were detached using Trypsin-EDTA (Amimed). Chondrocyte stimulation experiments were performedin either complete DMEM medium (Gibco), (10% FBS and 1% Penicillin Streptomycin (Sigma)), or incomplete DMEM (1% Penicillin Streptomycin only). For chondrocytes crystal formation analysis, cells were cultivated as described above for BMDM.

### Calcium phosphate crystal stimulation

BMDM and chondrocytes were left overnight in cell culture plates to allow adherence to the plate surface in complete DMEM. For experiments that required priming, cells were stimulated overnight with TLR-ligand, Pam3Cys (100ng/ml). The next day, the media was exchanged and the experiment was continued with incomplete DMEM or incomplete BGJb. Cells were stimulated or not with sterile HA crystals at 500 μg/ml and treated or not with STS 25mM for the indicated time. The supernatants were collected for cytokine ELISA and cells were placed in TRIzol (Life Technologies) for Real time PCR analysis (qRT-PCR).

### Crystal detection from BMDM and chondrocyte cultures

BMDM and chondrocytes were primed with Pam3Cys (100ng/ml) in complete BGJb 10% FBS, 50 μg/ml L-ascorbic acid 2-phosphate, 20 mM β-glycerol phosphate and 1% Penicillin Streptomycin). The next day, medium was exchanged and cells treated or not, with STS at different concentrations (0.2mM, 1mM, 5mM, or 25mM) in complete BGJb. After 1, 3 or 7 days, supernatant was collected for ELISA and LDH measurement, and cells were washed in PBS, fixed and crystal deposition analyzed through Alizarin red staining and quantification [27] or Von Kossa staining as described before [28].

### Real time PCR analysis

RNA was extracted (RNA Clean & Concentrator5-Zymoresearch), reverse transcribed (Superscript II- Invitrogen^™^), and quantitative Real Time PCR (qRT-PCR) with gene specific primers using the LightCycler480^®^system (Roche Applied Science) was performed (Table 1). Data was normalized against *Tbp* and *Gapdh* references genes, with fold induction of transcripts calculated against the unstimulated (Nt) control cells.

**Table 1:** Gene specific primers for qRT-PCR.

Gene	Forward primer (5’ → 3’)	Reverse primer (5’→ 3’)
*Adamts-4*	GCC CGA GTC CCA TTT CCC GC	GCC ATA ACC GTC AGC AGG TAG CG
*Adamts-5*	GAC AGA CCT ACG ATG CCA CCC AGC	ATG AGC GAG AAC ACT GAC CCC AGG
*Ank*	TGT CAA CCT CTT CGT GTC CC	GAC AAA ACA GAG CGT CAG CG
*Anx5*	CCT CAC GAC TCT ACG ATG CC	AGC CTG GAA CAA TGC CTG AG
*Coll2*	ACA CTT TCC AAC CGC AGT CA	GGG AGG ACG GTT GGG TAT CA
*Coll10*	AAA CGC CCA CAG GCA TAA AG	CAA CCC TGG CTC TCC TTG G
*Gapdh*	CTC ATG ACC ACA GTC CAT GC	CAC ATT GGG GGT AGG AAC AC
*Il-6*	TCC AGT TGC CTT CTT GGG AC	GTG TAA TTA AGC CTC CGA CT
*Pc-1*	CTG GTT TTG TCA GTA TGT GTG CT	CTC ACC GCA CCT GAA TTT GTT
*Pit-1*	CTC TCC GCT GCT TTC TGG TA	AGA GGT TGA TTC CGA TTG TGC
*Pit-2*	AAA CGC TAA TGG CTG GGG AA	AAC CAG GAG GCG ACA ATC TT
*Runx2*	GGG AAC CAA GAA GGC ACA GA	TGG AGT GGA TGG ATG GGG AT
*Sox9*	AAG ACT CTG GGC AAG CTC TGG A	TTG TCC GTT CTT CAC CGA CTT CCT
*Tbp*	CTT GAA ATC ATC CCT GCG AG	CGC TTT CAT TAA ATT CTT GAT GGT C
*Tnap*	TTG TGC CAG AGA AAG AGA GAG	GTT TCA GGG CAT TTT TCA AGG T

### Human cartilage explants experiments

Macroscopically intact knee cartilage from femoral condyles was obtained from 3 OA patients (Kellgren-Lawrence score of 4, mean age 74±14 years) from the Orthopedics Department (DAL, CHUV, Lausanne-CH) at time of joint replacement. Briefly, 6 mm diameter disks (9–15 disks/patient) were obtained from cartilage using a dermal punch. In order to match for location across treatment groups, each disk was divided in two equal parts, and each half was stimulated for 24h in individual 96 wells, coated with Poly(2-hydroxyethyl methacrylate) in culture medium (DMEM + 1% Penicillin Streptomycin + 50μg/ml of L-ascorbic acid 2-phosphate). Explants were stimulated with 500μg/ml of HA crystals in presence or absence of STS 25mM. The following comparisons were performed: unstimulated vs HA; HA vs HA+STS; unstimulated vs STS. At the end of the incubation period, supernatants were collected for IL-6 measurement by ELISA and IL-6 secretion normalized by the tissue weight.

### LDH measurement

LDH in supernatant was measured using CytoTox-ONE^™^ Homogeneous Membrane Integrity Assay (Promega) according to the manufacturer’s instructions. LDH release (%) was calculated by using the following formula. LDH release (%) = [(value in sample)—(background)] / [(value in Triton X-100-treated sample)—(background)] x100.

### Cytokine and chemokine quantification

At the reported time points of the different experiments, cell supernatants were assayed using murine or human IL-6 and murine TNF-α, MCP-1 or IL-1β ELISA kits (eBioscience). The manufacturer’s protocols were explicitly followed, and the results were read at 450nm using the Spectrax M5e (Molecular devices).

### ROS level measurement

Cytoplasmic ROS level was measured with dihydroethidium (DHE, Life Technologies), a superoxide indicator which exhibits blue-fluorescence in the cytosol until oxidized and that stains cell’s nucleus in bright fluorescent red when it intercalates within its DNA. Mitochondrial ROS level was measured with Red Mitochondrial Superoxide Indicator (MitoSOX, Life Technologies), that produces red fluorescence once is oxidized by superoxide. Briefly, chondrocytes in half area 96-well clear bottom black plate were stimulated with HA crystals (500 μg/ml) and treated or not with STS (25mM) for 1 hour. After stimulation, cells were loaded 30 min with DHE or MitoSOX, and fluorescence intensity was measured at 518 nm (excitation) and 605 nm (emission) and 510 nm (excitation) and 580 nm (emission) respectively using the Spectrax M5e (Molecular devices).

### Ethics statements

Experiments in mice were performed in strict accordance to the Swiss Federal Regulations. The protocol was approved by the “*Service de la consommation et des affaires vétérinaires du Canton de Vaud*”, Switzerland. All efforts were made to minimize suffering

Human samples were obtained with the approval of the Centre Hospitalier Universitaire Vaudois ethical committee and patients written informed consent.

### Statistical analysis

All values are expressed as the mean±SD. Variation between data sets was evaluated using the Student’s t test or one-way or two-way ANOVA test, where appropriate. Differences were considered statistically significant for a value of p<0.05. Data was analysed with GraphPad Prism software (GraphPad software), San Diego, CA.

## Results

### STS inhibits mineralization and reduces expression of annexin V by murine joint chondrocytes

**Fig 1:**
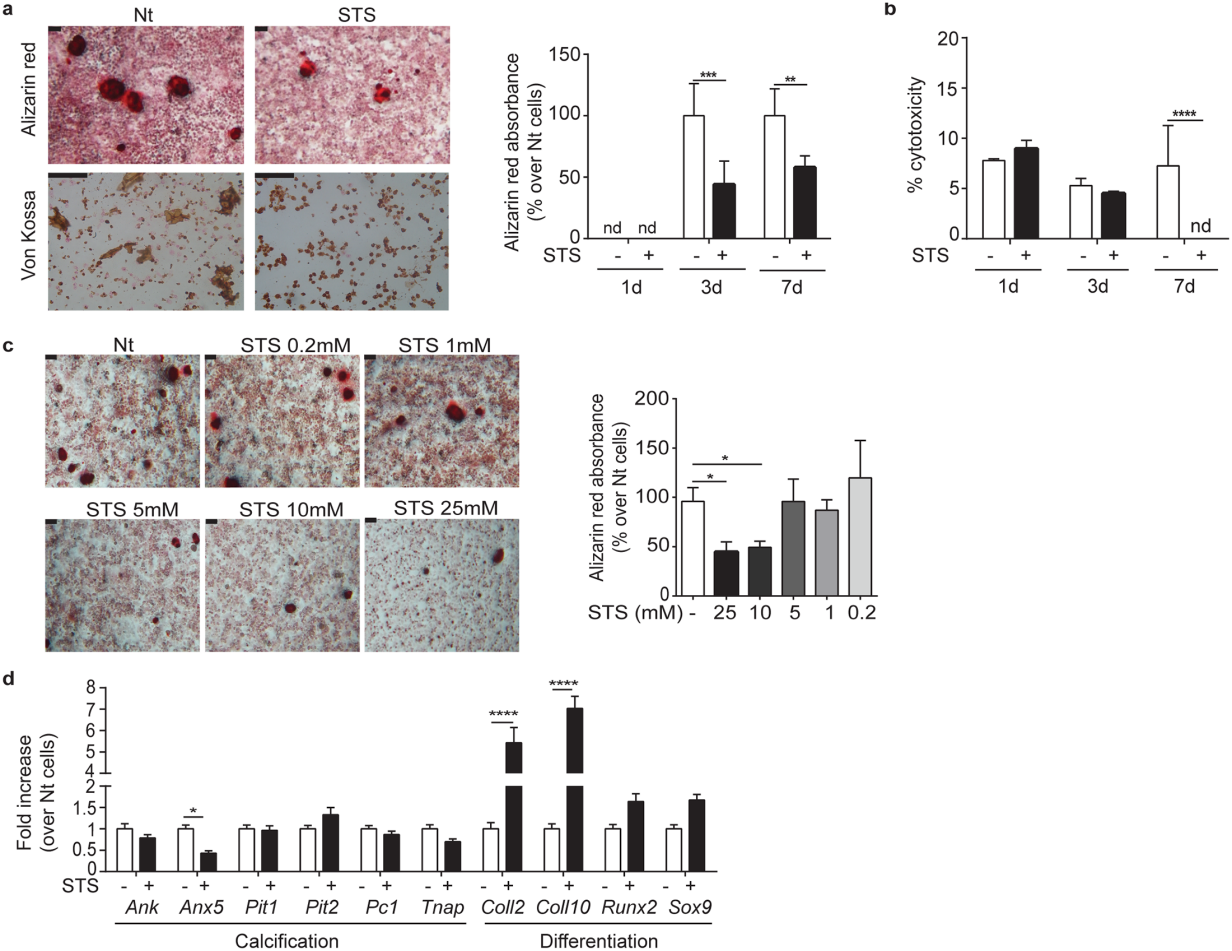
STS inhibits murine joint chondrocytes mineralization. (**a**) Alizarin red and Von Kossa staining of murine chondrocytes treated or not (Nt) with 25mM STS for 7 days in complete BGJb. Pictures represent one representative culture well of one experiment (five independent experiments were performed). Scale bar 100μm. The graph shows Alizarin red absorbance at 405nm, expressed in % over Nt cells at 1, 3 or 7 days of culture. Values represent means±SD of triplicates from one representative experiment of three independent experiments. (**b**) Percentage of cytotoxicity in murine chondrocytes treated or not with 25mM STS for 1, 3 or 7 days. Values represent means±SD of triplicates from one representative experiment of three independent experiments. (**c**) Alizarin red staining of murine chondrocytes treated or not (Nt) with different concentrations of STS for 7 days. Pictures represent triplicates from one experiment. Scale bar 100μm. The graph shows Alizarin red absorbance at 405nm, expressed in % over Nt cells at 7 days of culture. Values represent means±SD of triplicates from one representative experiment of three independent experiments. (**d**) qRT-PCR of the indicated genes in murine chondrocytes treated or not with 25 mM STS for 7 days in complete DMEM. Values represent means±SD of triplicate samples. *p<0.05, ** *p*<0.01, *** *p*<0.001, **** *p*<0.0001. nd = not detectable

We first evaluated STS's effects on chondrocyte mineralization. Primary murine joint chondrocytes were allowed to calcify in the presence of calcifying medium and calcification was assessed at different days: 1, 3 and 7. Calcium crystals were detected at both day 3 and day 7 by Alizarin red and Von Kossa staining (Fig 1a, pictures). As a non-mineralizing negative control cell type, we used murine BMDM, which in the same conditions did not produce crystals (data not shown). In chondrocyte cultures, 25mM STS treatment resulted in a clear reduction in crystal deposits (Fig 1a, pictures). Spectrophotometric quantification of Alizarin red staining, after crystal acidic extraction from the entire cell monolayer, confirmed that STS inhibited by 50% chondrocyte calcification at day 3 and 7 (Fig 1a, graph). This effect of STS is not due to cytotoxicity, as measured by LDH activity (Fig 1b). The effect of STS on chondrocyte mineralization was dose-dependent from 0.2mM to 25mM as shown by decreased Alizarin-red absorbance (Fig 1c). To characterize how STS might interfere with mineralization in chondrocyte cultures, we assessed changes in the expression of genes involved in the calcification process (*Ank*, *Anx5*, *Pit1*, *Pit2*, *Pc-1*, *Tnap*) by real-time PCR after 7 days in culture in the presence or the absence of 25mM STS. Results in Fig 1d show that *Anx5* expression diminished two fold in the presence of 25mM STS. In contrast, at the same concentration, STS did not significantly alter the expression levels of the other genes. Thus, STS inhibited calcification and the expression of *Anx5* in cultured joint chondrocytes.

### STS alters some aspects of chondrocyte differentiation

Chondrocyte differentiation is characterized by coordinated changes of gene expression from early chondrocytes (expressing type 2 collagen/*Coll2* and *Sox9*) to hypertrophic and terminally differentiated mineralizing chondrocytes (expressing type 10 collagen/*Coll10* and *Runx2*). We wondered if STS reduces BCP crystal formation by blocking chondrocyte terminal differentiation. To test this hypothesis, chondrocytes were cultured for 7 days in the presence or in the absence of STS and expression levels of *Coll2*, *Coll10*, *Sox9* and *Runx2* were then assessed by quantitative PCR. Results in Fig 1d show that STS induced five-fold up-regulation of *Coll2* and seven-fold up-regulation of *Coll10*, without altering *Runx2* or *Sox9* expression levels. Therefore, STS altered expression of some chondrocyte differentiation markers *in vitro*, but it failed to recapitulate the coordinated changes in expression that are typical of chondrocyte differentiation (*Coll2* and *Sox9* down-regulation, *Runx2* and *Coll10* up-regulation).

### STS inhibits both IL-6 production as well as downstream MCP-1 production in chondrocytes

**Fig 2:**
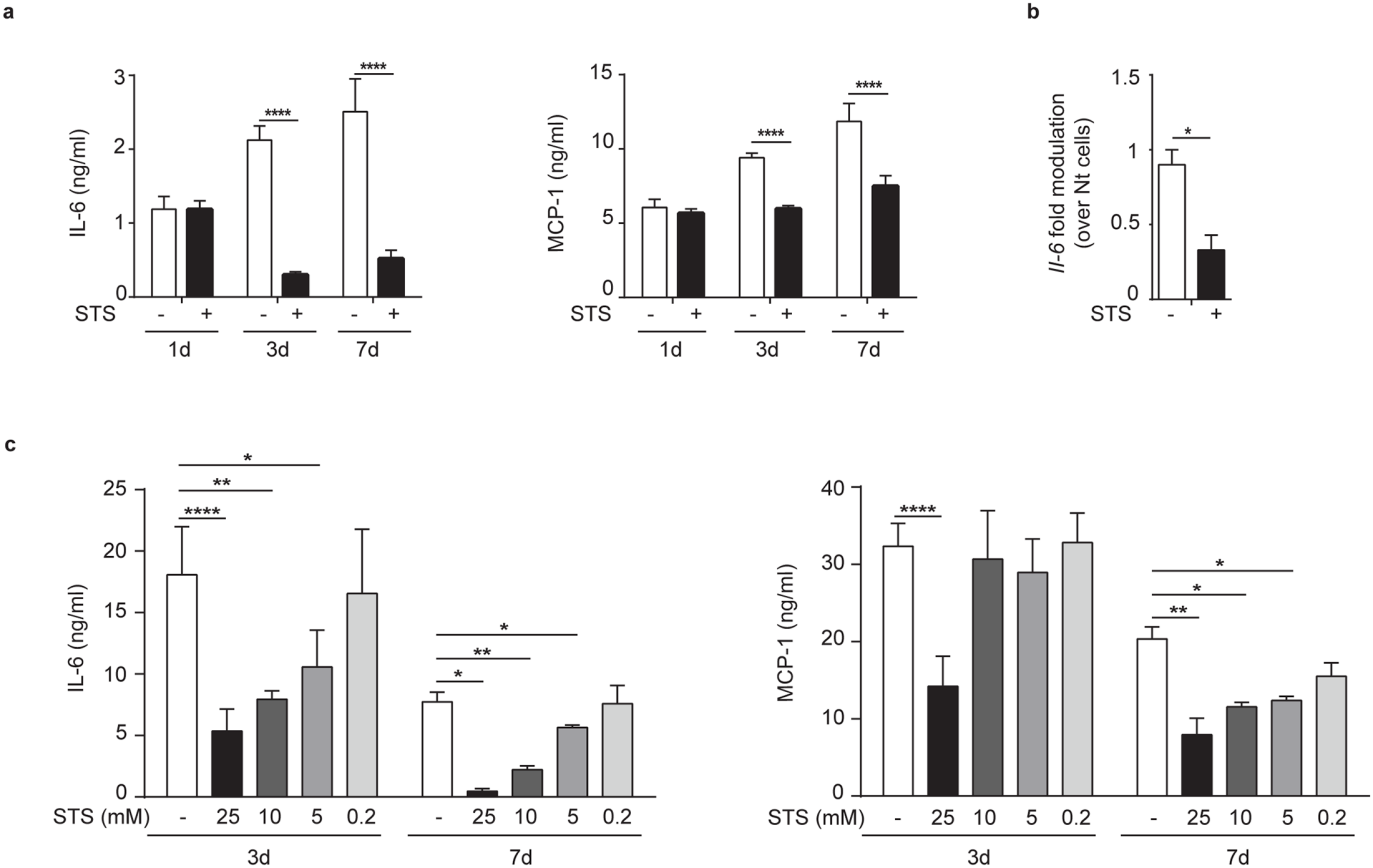
STS inhibits chondrocyte IL-6 and MCP-1 secretion in a dose-dependent manner. (**a**) IL-6 and MCP-1 secretion by primed murine joint chondrocytes treated or not with 25mM STS for 1, 3 or 7 days. Values represent means±SD of triplicates from one representative experiment of three independent experiments. (**b**) qRT-PCR of IL-6 gene in primed murine chondrocytes treated or not with 25 mM STS for 3 days. (**c**) IL-6 and MCP-1 secretion by primed murine joint chondrocytes treated or not with different concentrations of STS for 3 and 7 days. Values represent means±SD of triplicates from one experiment. *p<0.05, ** *p*<0.01, *** *p*<0.001, **** *p*<0.0001.

We recently demonstrated that BCP crystals can induce IL-6 production in chondrocytes and that IL-6 can, in turn, promote mineralization [14]. We therefore speculated that STS, in addition to inhibiting crystals formation, could also affect IL-6 and IL-6-induced MCP-1 secretion in chondrocytes. We found that STS inhibited ten times IL-6 secretion and two times MCP-1 secretion, when crystals were present in chondrocyte cultures. In contrast, STS had no effect on IL-6 secretion at day 1, when no crystals were seen in chondrocytes culture (Fig 2a). The inhibitory effect of STS on chondrocyte IL-6 secretion was accompanied by three-fold decreased IL-6 mRNA level, as shown by results in Fig 2b. Finally, STS's effects on IL-6 and MCP-1 production were dose-dependent from 0.2mM to 25mM after 3 days or 7 days of culture (Fig 2c). Altogether, these results showed that both IL-6 production and downstream IL-6 mediated effects are inhibited by the presence of STS.

### STS inhibits HA-induced IL-6 and MCP-1 secretion in chondrocytes and human cartilage explants but not in BMDM

**Fig 3:**
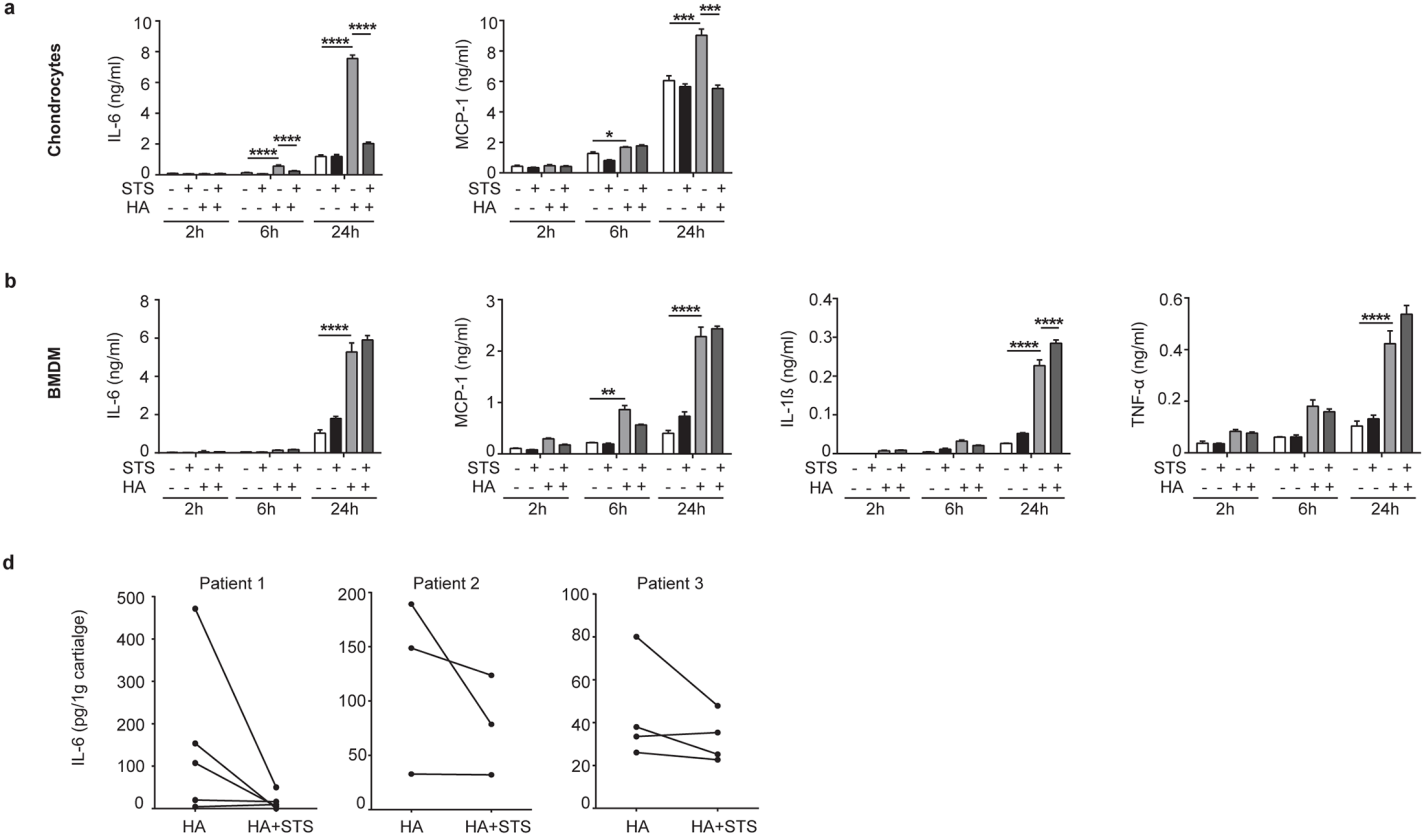
STS inhibits HA-induced cytokine and chemokine selectively in chondrocytes. (**a**) IL-6 and MCP-1 secretion by primed murine joint chondrocytes and (**b**) IL-6, MCP-1, IL-1β and TNF-α secretion by primed murine BMDM, stimulated or not with HA crystals (500ug/ml) and treated or not with 25mM STS 25mM for 2, 6 and 24hrs. Values represent means±SD of triplicates from one representative experiment of three independent experiments. (**c**) IL-6 secretion by human cartilage explants was measured by ELISA. Explants were stimulated with 500μg/ml of HA crystals in presence or absence of STS 25Mm for 24 h. Matched-halves of cartilage explants are connected by a line (5 explants for patients 1, 3 for patient 2, 4 for patient 3). Values represent means±SD of triplicate samples. *p<0.05, ** *p*<0.01, *** *p*<0.001, **** *p*<0.0001.

In order to investigate if STS effects are cell-specific, we stimulated both mineralizing competent cells (chondrocytes) and non-mineralizing cells (BMDM) with exogenous HA crystals in the presence or in the absence of STS. As previously demonstrated by our group [14], HA crystals significantly increased IL-6 and MCP-1 secretion in chondrocytes, after 6 hours of stimulation (Fig 3a). When present for 6h or 24h at 25mM, STS was able to reduce both HA-induced IL-6 and MCP-1 production (Fig 3a) to control levels. STS was also able to significantly diminish CPPD-induced IL-6 and MCP-1 at 24 hours (Data not shown). By contrast, STS failed to inhibit HA-induced IL-6, IL-1β, TNF-α and MCP-1 in BMDM (Fig 3b). Finally, in both cell types, STS had no measurable effect on the basal secretion level of the analyzed cytokines and chemokine (Fig 3a and 3b). We have previously demonstrated, using human cartilage explants, that HA crystals induced IL-6 secretion [14]. We speculated that STS could inhibit HA-induced IL-6 in this setting as well. In fact, STS antagonized, although at different levels, the effects of HA crystals on IL-6 secretion by human cartilage explants (Fig 3c).

### STS inhibits HA-induced ROS generation and HA-induced matrix-degrading enzymes in chondrocytes

**Fig 4:**
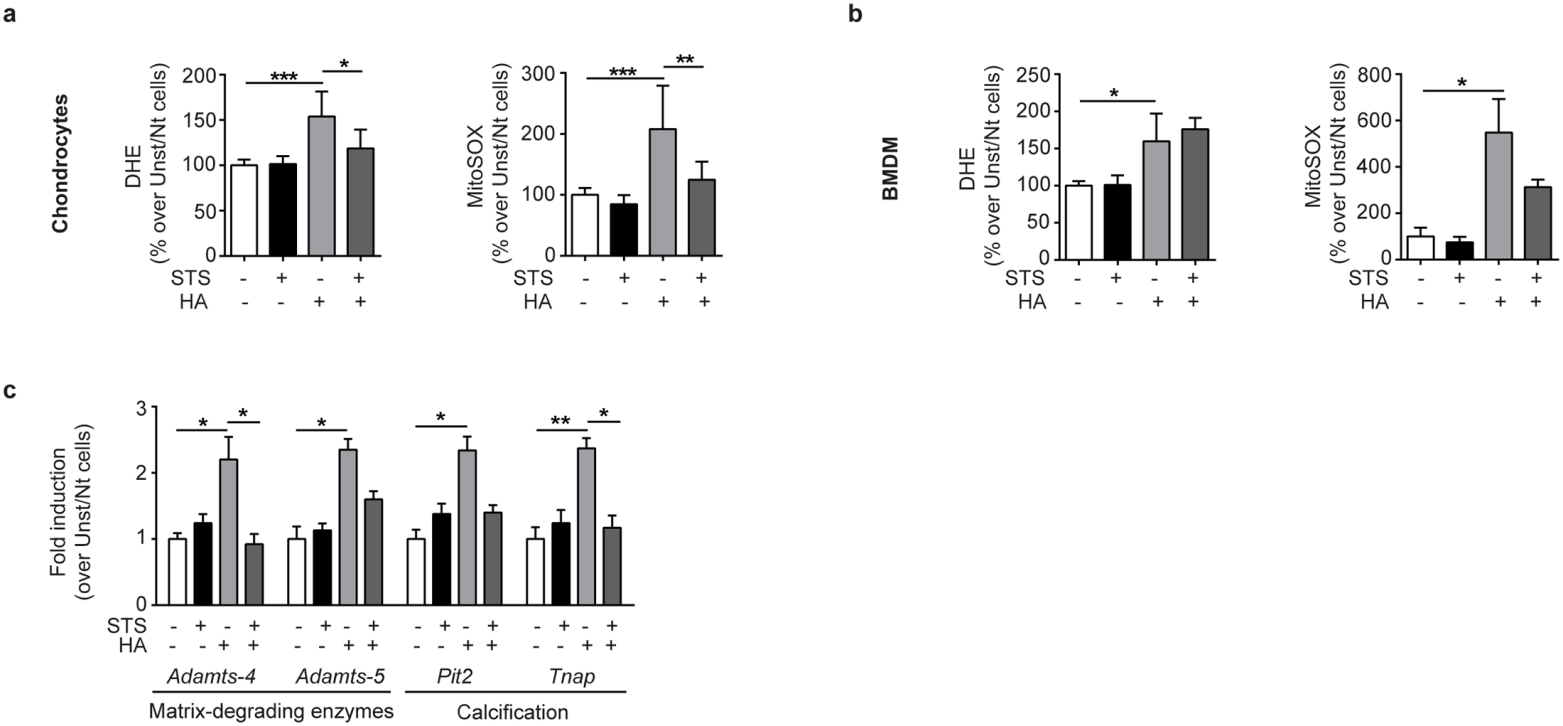
STS inhibits HA-induced ROS production and enzymes involved in cartilage degradation. ROS generation by (**a**) murine joint chondrocytes and (**b**) BMDM stimulated or not with HA crystals (500ug/ml) and treated or not with 25mM STS for 1h. Dihydroethidium (DHE) staining was used as an indicator of intracellular ROS generation. MitoSOX^™^ Red reagent was used as an indicator of mitochondrial ROS generation. Values represent means±SD of triplicates from one representative experiment of two independent experiments and are expressed as %of ROS in unstimulated cells. (**c**) qRT-PCR of the indicated genes in murine chondrocytes stimulated or not with HA crystals (500ug/ml) and treated or not with 25mM STS for 30 minutes in DMEM. Values represent means±SD of triplicate samples. *p<0.05, ** *p*<0.01, *** *p*<0.001, **** *p*<0.0001.

As we previously reported that HA crystals induced cellular ROS [29], we asked if STS was able to block not only cytokine and chemokine secretion but also ROS production. Indeed, in chondrocytes we found that STS significantly inhibited, at the control level, both mitochondrial (detected by the MitoSOX) and cytoplasmic (detected by DHE, a fluorescent superoxide indicator) ROS generated upon HA crystals stimulation (Fig 4a). In line with the selective inhibition of cytokine/chemokine in chondrocytes but not in BMDM, STS had no statistically significant antioxidant effect in BMDM (Fig 4b). Finally, qRT-PCR analysis revealed that STS significantly antagonized calcium-contaning crystals effect on gene expression. In particular, STS reversed HA-induced two-fold up-regulation of genes coding for catabolic enzymes (*Adamts-4* and *-5*), and of genes involved in the calcification process (*Pit2* and *Tnap*) (Fig 4c), although only the inhibition of HA-induced expression of *Adamts-4* and *Tnap* reached statistical significance.

### STS inhibits formation of periarticular calcific deposits and cartilage damage in experimental OA

**Fig 5:**
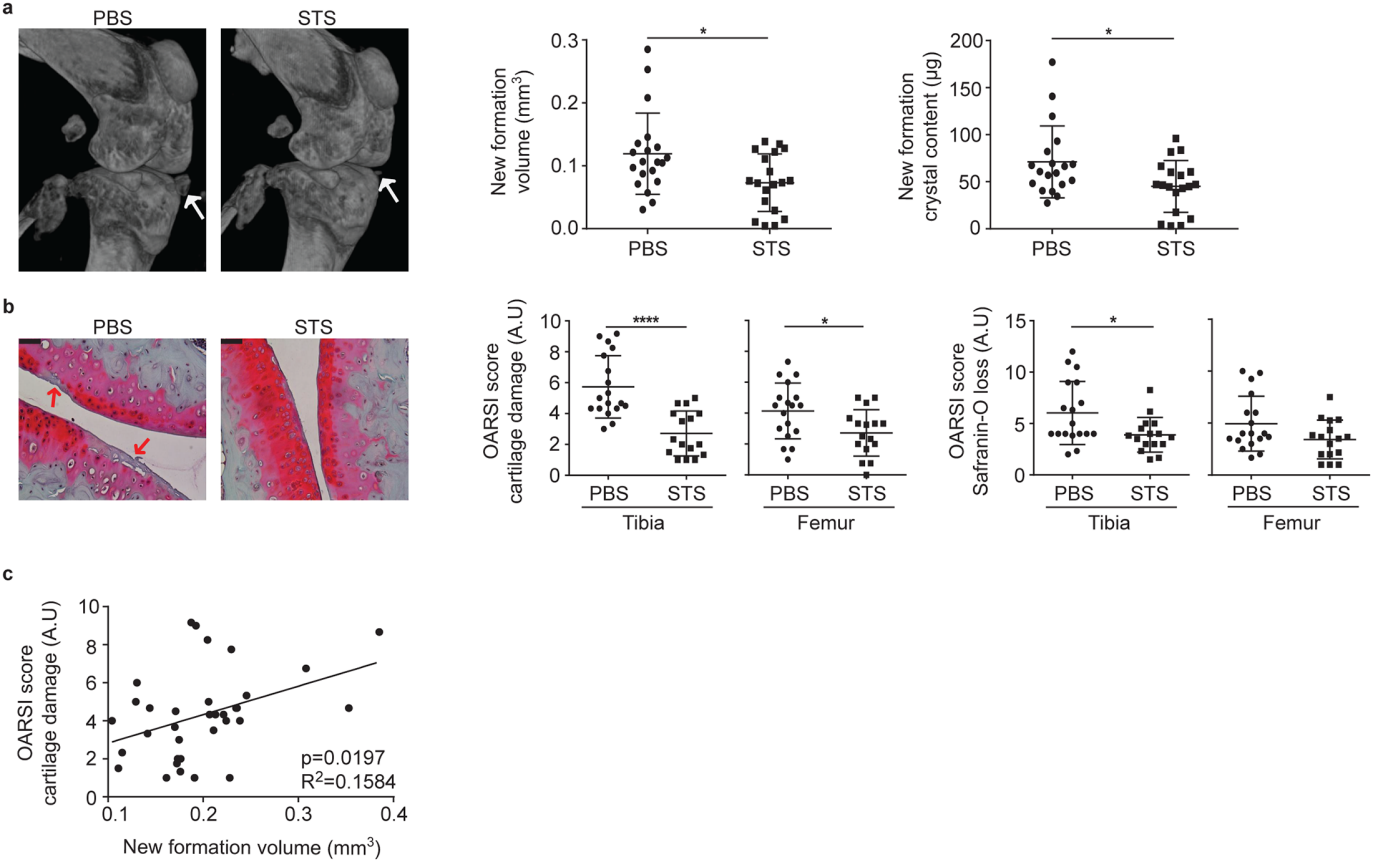
STS inhibits formation of new calcific deposits and cartilage degradation in experimental OA. (**a**) Representative micro-CT scan images of menisectomized murine knee joints after vehicle (PBS) or STS treatment for two months after surgery. White arrows show periarticular deposits in menisectomized knees treated with vehicle and their reduction in STS treated mice. Graphs show CTAnalyzer quantitative analysis of new formation volume (mm^3^) and new formation crystal content (μg) in PBS- and STS-treated menisectomized mice. Data are expressed as the mean±SD (**b**) Representative histologies of PBS- and STS-treated menisectomized knees, stained with Safranin-O. Red arrows show degenerative OA changes in the articular cartilage of PBS-treated mice. Scale bars 150 μm. Graphs show tibial and femoral scoring of cartilage damage and Safranin-O loss, accordingly to OARSI method. Values represent means±SD. (**c**) Correlation graph between tibial cartilage damage score and new formation volume of pooled PBS- and STS-treated mice. (Mice number: PBS n = 20, STS n = 19).

As calcium crystal deposition in cartilage and around the joint is linked to human OA, we examined if calcific deposits and cartilage degradation are linked in experimental OA. We also investigated if the administration of STS had an effect on experimental OA. Mice were subjected to knee menisectomy (MNX) and treated with STS or PBS as vehicle. Then a microCT-scan examination was conducted 8 weeks after the beginning of treatment. Pictures on Fig 5a show that STS administration markedly inhibited new calcific formations in knee joint. In contrast, bone morphometric parameters appeared similar in PBS- and STS-treated mice (results not shown). In line with micro-CT findings, the volume of newly formed crystals, as well as the overall newly formed crystal content, was significantly decreased in STS-treated mice by 25% (Fig 5a, graphs). In addition, the effect of STS treatment on cartilage damage and proteoglycan content was assessed in meniscectomized mice by Safranin-O staining, and quantified by OARSI scoring. Pictures in Fig 5b show that PBS-treated mice are affected by cartilage fissuration and fibrillation (red arrows), which were reduced by up to 40% by STS-treatment. Likewise, OARSI scoring revealed that STS treatment prevents cartilage damage and proteoglycan loss although statistical significance was only reached for tibial cartilage. Interestingly, there was a statistically significant positive correlation between the volume of the new mineralized structures and tibial cartilage degradation score, strongly suggesting that these new deposits have an etiologic significance in OA (Fig 5c).

## Discussion

We have here provided evidence that STS significantly reduced calcium-containing crystal formation both in cultured murine chondrocytes and in OA mice, thus highlighting the therapeutic potential of this agent as a disease-modifying drug for crystal-associated OA. *In vitro*, STS inhibited by up to 50% chondrocyte mineralization, as showed by quantification of Alizarin red and Von Kossa staining. The inhibitory effect of STS on crystal formation was confirmed i*n vivo*, in menisectomy-induced OA: indeed, STS administration decreased the volume and the crystal content of the new calcific deposits formed within the joint after MNX, and attenuated tibial and femoral cartilage degradation as well as proteoglycan loss. In addition, we found a significant positive correlation between the size of the calcific deposits and cartilage degradation score, further strengthening the notion that BCP crystals serve a pathogenic role in OA [13]. In keeping with its anti-mineralization effect, STS significantly reduces the expression levels of *Anx5*, a collagen-regulated calcium channel involved in matrix vesicle-initiated cartilage calcification [30, 31]. This result suggests that the observed STS-driven reduction in calcification might be the consequence of reduced calcium uptake into matrix vesicles leading to inhibition of BCP crystals nucleation. In line with our hypothesis, Kirsch and colleagues [32] showed that *Anx5* overexpression promotes calcification in chondrocytes and that *Anx5* down modulation by the corresponding siRNA led to inhibition of mineralization [32]. Accordingly, during pathological calcification in human OA, cartilage showed significant upregulation of *Anx5* [33] and increased release of matrix vescicles containing Anx5 [34]. By contrast, *in vivo* studies in mice show normal growth plate, bone architecture and matrix vescicles distribution in Anx5^-/-^ and Anx5^-/-^Anx6^-/-^ mice [35]. Given those discrepancies, more experiments are warranted to substantiate our hypothesis. Regardless, our results favor this hypothesis over the notion that STS reduces BCP crystal formation by globally altering chondrocyte differentiation status. Indeed, whereas STS increased both *Coll2* and *Coll10* gene expression, it failed to modulate *Sox9* or *Runx2*, two markers of early and terminal chondrocytic differentiation respectively. Thus, STS effect on mineralization cannot simply be explained in terms of a global chondrocytic differentiation blockade.

In addition to its anti-mineralizing role, STS has clear anti-inflammatory effects as demonstrated by inhibition of crystal-induced IL-6 and MCP-1 in chondrocytes. Our results are in line with a previous report describing anti-inflammatory properties of comparable STS concentrations, in LPS or TNF-α-induced cytokine production by endothelial cells [36]. In agreement with its *in vitro* anti-inflammatory properties, STS administration in mice improved survival after endotoxemia [37, 38] and acute liver failure [39]. Likewise, STS attenuated both acute lung injury [36] and ischemic brain injury [40]. Furthermore, our results demonstrated that STS has anti-catabolic effect since it was able to inhibit HA-induced *Adamts-4* and *5* up-regulation

Previous reports have established that IL-6 production is linked to OA severity: intra-articular IL-6 injection causing OA cartilage destruction, whereas neutralization of IL-6 inhibits cartilage destruction in OA mouse models [41]. Likewise, in the menisectomy (MNX) model of OA, cartilage from IL-6 knockout mice was protected from degradation when compared to that of wild type mice [41]. Using the same MNX model, we found that STS treatment inhibited periarticular calcification and attenuated cartilage degradation in mice. STS protective effect on cartilage degradation could be accounted for by its inhibitory effects on chondrocyte IL-6 secretion. Indeed, in chondrocytes, IL-6 shows both promineralizing [14] and catabolic activity via *ADAMTs* up-regulation [42], and we have demonstrated that STS was able to inhibit both effects. Whether inhibition of IL-6 expression by STS is the main mechanism involved in cartilage protection, or whether alternative modes of action mediate STS beneficial effects remains to be established.

Thiosulfate (S_2_O_3_^2-^) is a well-known intermediate in the oxidative hydrogen sulfide (H_2_S) metabolism into sulfite and sulfate. Within cells, S_2_O_3_^2-^ and H_2_S co-exist in a dynamic oxido-reductive equilibrium that is subjected to a complex cell- and context-specific regulation [43]. Fox et al. recently showed that endogenous H_2_S production plays an antioxidant cytoprotective role in mesenchymal cells cultures [44]. Although we did not monitor H_2_S production in response to *in vitro* or *in vivo* STS treatment, it is likely that at least part of the antioxidant effect of exogenous STS reported herein is linked to increased H_2_S signaling. We indeed demonstrated that STS inhibits HA-induced ROS production by chondrocytes. Further studies are needed to investigate whether the underlying mechanism of this effect is due to STS modulation of ROS generating enzymes or of antioxidant enzymes. Interestingly, published studies demonstrated that ROS are in turn implied in calcification. They modulate initiation of the hypertrophic changes in chondrocytes [45] and they induce calcification of human dental pulp cells [46]. Agharazii et al. showed a relationship between ROS and arterial calcification in chronic kidney disease patients [47]. We will therefore investigate whether ROS are able to induce calcification in our chondrocyte cultures and if STS is able to block this effect. *In vivo*, STS might therefore be active both upstream and downstream to IL-6R signaling by modulating IL-6 production and preventing resulting oxydative stress respectively [48]. In this context, the apparent cell selectivity of STS treatment (inhibitory in chondrocytes but not in macrophages), could relate to lineage specific differences in ROS production, S_2_O_3_^2-^ transport and/or H_2_S homeostasis.

Of note, the doses of STS used in our study, both *in vitro* (25mM) and *in vivo* (0.4g/Kg) were very similar to those reported before in *in vitro* [36], preclinical [20] [36] and clinical studies [49–51], where efficacy was reached with low cytotoxicity and side effects. In our experiments we have used STS in a preventive way, at time of OA induction. Future experiments will be performed to verify that STS can act not only in preventive, but also in a curative approach. In conclusion, our results reveal a novel beneficial role of STS in osteoarthritis, which may lead to the development of new therapeutics aimed at preventing or reducing intra-articular calcifications and disease progression.

## Abbreviations

OA: osteoarthritis
STS: sodium thiosulfate
BCP: basic calcium phosphate
CPPD: calcium pyrophosphate dihydrate
MNX: menisectomy
